# Induction of necrosis symptoms by potato virus X in AGO2-silenced tomato plants associates with reduced transcript accumulation of copper chaperon for superoxide dismutase gene

**DOI:** 10.1016/j.virusres.2024.199436

**Published:** 2024-07-18

**Authors:** Joon Kwon, Kento Mori, Tetsuo Maoka, Teruo Sano, Kenji S. Nakahara

**Affiliations:** a Graduate School of Agriculture, Hokkaido University, Sapporo, Hokkaido 060-8589, Japan; b Institute for Plant Protection, National Agriculture and Food Research Organization (NIPP, NARO), Tsukuba, Ibaraki, 305-8666, Japan; c Faculty of Agriculture and Life Science, Hirosaki University, Hirosaki 036-8561, Japan; d Research Faculty of Agriculture, Hokkaido University, Sapporo, Hokkaido 060-8589, Japan

**Keywords:** Systemic necrosis, Argonaute (AGO)2-knockdown tomato, Potato virus X (PVX), MicroRNA 398 (miR398), Copper chaperon for superoxide dismutase (CCS)1, Reactive oxygen species (ROS)

## Abstract

RNA silencing is a prominent antiviral defense mechanism in plants. When infected with a virus, RNA silencing–deficient plants tend to show exacerbated symptoms along with increased virus accumulation. However, how symptoms are exacerbated is little understood. Here, we investigated the role of the copper chaperon for superoxide dismutase (CCS) 1, in systemic necrosis observed in Argonaute (AGO)2-silenced tomato plants infected with potato virus X (PVX). While infection with the UK3 strain of PVX induced mosaic symptoms in tomato plants, systemic necrosis occurred when AGO2 was silenced. The *CCS1* mRNA level was reduced and micro RNA398 (*miR398), which potentially target CCS1,* was increased in AGO2-knockdown tomato plants infected with PVX-UK3. Ectopic expression of *CCS1* using recombinant PVX attenuated necrosis, suggesting that CCS1 alleviates systemic necrosis by activating superoxide dismutases to scavenge reactive oxygen species. Previous reports have indicated a decrease in the levels of CCS1 and superoxide dismutases along with an increased level of miR398 in plants infected with other viruses and viroids, and thus might represent shared regulatory mechanisms that exacerbate symptoms in these plants.

## Highlights

• AGO2-deficient plants infected with potato virus X show systemic necrosis.
• Systemic necrosis was associated with reduction of the *CCS1* mRNA level.
• Genetic proof implicates CCS1 in systemic necrosis.

## 1 Introduction

Virus infection in plants does not always induce symptoms; symptom severity depends mainly on interactions between the virus and plant species, and on environmental conditions (Van Loon, 1997; Pagán et al., 2007; González et al., 2021). Slight variations in plant or viral genomes may alter symptom severity. How viral infections cause diverse symptoms are not fully understood. Understanding the molecular mechanisms of and controlling severe symptoms such as systemic necrosis caused by infection with highly virulent viruses are important for crop production since severe symptoms result in devastating losses of crop commercial value or even total yield loss.

Systemic necrosis induced by virus infections has been ascribed to hypersensitive response–mediated programmed cell death (HR-PCD) (Greenberg, 1997; Jacobson et al., 1997; McDowell and Dangl*,* 2000). It is often part of the defense mechanism against pathogen attack via HR. HR-PCD is generally triggered by the recognition of a protein encoded by the invading virus via a plant resistance (*R*) gene, most of which encode nucleotide-binding, leucine-rich-repeat (NB-LRR) proteins. HR-PCD is generally effective against invading viruses and other biotrophic plant pathogens because they require living cells for infection. However, when a protein encoded by an *R* gene recognizes an invading virus but the triggered HR-PCD fails to restrict infection, the virus spreads systemically, accompanied by HR-PCD, resulting in systemic necrosis. Regardless of R gene mediated recognition and HR-PCD, there are also cases of viral infections with necrotic symptoms. These symptoms include those caused by the endoplasmic reticulum membrane, where the expression of viral proteins disrupts normal protein synthesis and cellular balance, promoting the ER stress and unfolded protein response (UPR) in host plants (Khurana et al., 2005; Williams and Dickman, 2008; Verchot, 2016; Bhattacharyya and Chakraborty, 2018; Aguilar et al., 2019). In previous study reported that *potexviruses, potyviruses* and *reovirus* (positive sense RNA viruses), caused high biosynthetic burden on the ER, this load also increases the likelihood of the accumulation of deformed proteins and this causes ER stress when homeostasis is destroyed, such as the accumulation of deformed proteins, and the reaction system called UPR is activated to provide alleviation (Ju et al., 2005; Grangeon et al., 2013; Williams et al., 2014).

RNA silencing is another major plant antiviral defense mechanism, and this is why defects in RNA silencing increase plant susceptibility to viruses and are associated with increased virus multiplication and more severe symptoms (Vance and Vaucheret, 2001; Wang and Metzlaff, 2005; Agius et al., 2012; Wang et al., 2012; Nakahara and Masuta, 2014; Akhtel et al., 2021; Leonetti et al., 2021). Recently, RNA silencing–defective tomato (cv. Moneymaker) plants were generated by knocking down (using double-stranded RNA–mediated RNA silencing) the genes encoding dicer-like proteins 2 and 4 (DCL2,4), which are key factors in small RNA (sRNA) biogenesis for RNA silencing (Suzuki et al., 2019). In this study, when inoculated with potato spindle tuber viroid (PSTVd), wild-type tomato plants allowed PSTVd to infect systemically but showed only weak symptoms, whereas PSTVd-infected *DCL2,4*-knockdown plants showed lethal systemic necrosis. The necrosis was associated with increased levels of microRNA398 (*miR398*) and decreased levels of its targets, the copper chaperon for superoxide dismutase (CCS) 1 and superoxide dismutases (SODs). Futhermore, it was found the excessive accumulation of reactive oxygen species (ROS) in necrotic tissues of infected *DCL2,4*-knockdown plants and implied that *miR398*, CCS1 and SOD dysregulation is responsible for the lethal systemic necrosis. SODs scavenge superoxide (O_2_^−^) by converting it into hydrogen peroxide (H_2_O_2_), and their regulation by miRNA398s is important for oxidative stress tolerance (Beauclair et al., 2010). ROS play a central role in plant immune responses and regulation of cell death and cell survival (Doke, 1983; Lamb and Dixon, 1997; Marchi et al., 2012).

In addition to DCL2,4-knockdown plants, we created RNA silencing–deficient tomato plants by silencing the RNA-dependent RNA polymerase 6 (*RDR6*) and Argonaute 2 (*AGO2*) genes (Kwon et al., 2020). Moneymaker tomato is susceptible to potato virus X (PVX), and mild mosaic and distortion symptoms develop on leaves of infected plants. PVX infection causes exacerbated symptoms in *DCL2,4*-knockdown plants and systemic necrosis in *AGO2*-knockdown plants (Kwon et al., 2020). Together with DCL and RDR, AGO is a core component of the RNA-silencing machinery responsible for slicing target RNA. *Arabidopsis thaliana* has 10 AGO homologues, of which AGO1, AGO2, and AGO7 are involved in antiviral defense (Garcia-Ruiz et al., 2015). In *A. thaliana*, AGO2 is critical for defense against PVX because the *AGO2*-knockout mutant but not col-0 (wild type) is infected systemically (Jaubert et al., 2011). Consistent with the observations in tomato (Kwon et al. 2020), *AGO2*-knockout *Nicotiana benthamiana* infected with PVX shows systemic necrosis, whereas wild-type *N. benthamiana* shows mosaic symptoms (Ludman et al., 2017). This necrosis depends on double-stranded RNA–binding protein 2 (Fátyol et al., 2020).

In this study, we examined host genes involved in systemic necrosis in *AGO2*-knockdown plants infected with PVX. Similar with observation for systemic necrosis in *DCL2,4*-knockdown plants infected with PSTVd (Suzuki et al., 2019), ROS accumulated and *miR398* levels increased while the expression of SOD genes decreased in plants with necrotic symptoms. Our results suggest that tomato CCS1 is responsible for systemic necrosis in tomato.

## 2 Materials and methods

### 2.1 Plants and viruses

**Fig. 1:**
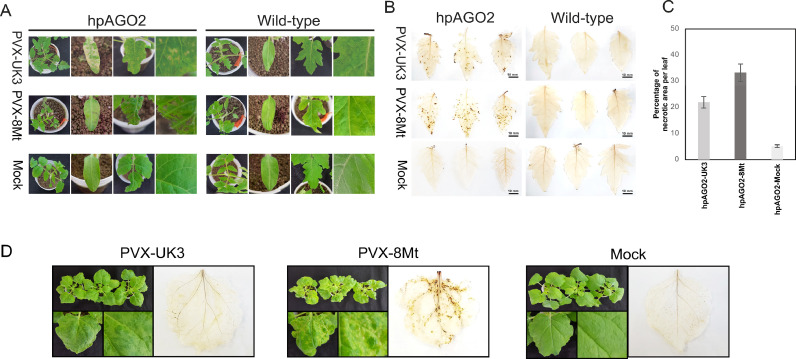
Reactions of tomato (cv. Moneymaker) and *Nicotiana benthamiana* plants following mechanical inoculation with PVX-UK3 or PVX-8Mt. (A) Symptoms developed in wild-type and AGO2-knockdown tomato plants (hpAGO2). Images were captured at 15 days-post-inoculation (dpi). Mock, inoculation with buffer. (B) Upper leaves stained with DAB at 15 dpi. (C) Graph showing comparison of the percentage of necrotic lesion on leaves from the above experiment. (D) Symptoms developed in inoculated *N. benthamiana*. Images of leaves and DAB staining were captured at 15 dpi.

**Fig. 2:**
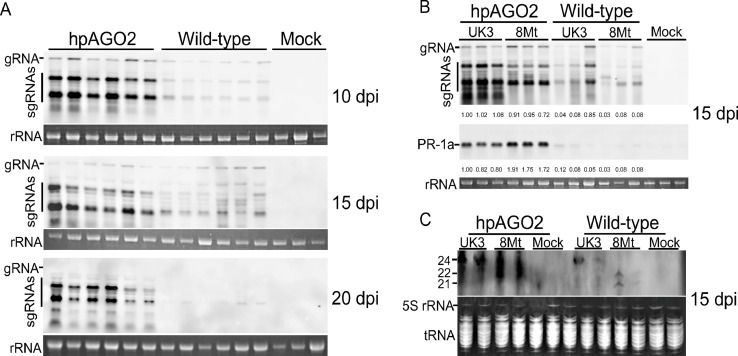
Northern blot analysis of total RNA extracted from upper leaves at 15 dpi. (A) Total RNA was extracted from non-inoculated upper leaves at the indicated time points from transgenic lines (hpAGO2) and wild-type tomato plants inoculated with PVX-UK3 or buffer (mock) and was fractionated by agarose gel electrophoresis. Northern blotting was performed with a DIG-PVX-cRNA probe. Ribosomal RNAs (rRNAs) were shown as loading controls stained with ethidium bromide (EtBr). gRNA, PVX genomic RNA; sgRNAs, subgenomic RNAs. (B) Total RNA was extracted at 15 dpi from non-inoculated upper leaves of transgenic lines (hpAGO2) and wild-type tomato plants inoculated with PVX-UK3 or PVX-8Mt and was fractionated by agarose gel electrophoresis to detect gRNA, sgRNAs, and the transcript of the *pathogenesis-related 1a* gene (*PR-1a*). rRNA stained with EtBr was used as a loading control. The ImageJ software was used for RNA expression level quantification. (C) Total RNA extracted at 15 dpi was fractionated by 12% PAGE in the presence of 8 M urea and northern blot analysis of small RNA from the corresponding samples in (B). tRNA and 5S RNA, loading controls stained with EtBr.

We previously created transgenic tomato plants (hpAGO2.3), in which double-stranded RNAs cognate to the tomato *AGO (SlAGO) 2* and *3* genes were expressed to silence these *SlAGO* genes, and analyzed their viral susceptibility (Kwon et al., 2020). Since the expression of SlAGO2 but not SlAGO3 was significantly suppressed in the hpAGO2.3 plants, we referred to them as SlAGO2-silenced plants and named them hpAGO2 in the purpose of this study. hpAGO2 plants were created from wild-type tomato (*Solanum lycopersicum* cv. Moneymaker) (Kwon et al., 2020). Wild tobacco plants, *N. benthamiana*, were also used. The binary vector pGR107, which contains the infectious cDNA of PVX (UK3 strain), was kindly provided by David C. Baulcombe (University of Cambridge, Cambridge, United Kingdom). Another PVX strain, which caused necrotic symptoms in infected tobacco plants, and was provided by the Yatsugatake branch farm of the Center for Seeds and Seedlings, National Agriculture and Food Research Organization (NCSS) in Japan. The strain was then maintained at the Hokkaido Agricultural Research Center of the National Agriculture and Food Research Organization (NARO). This strain, designated as PVX-8Mt, was used for inoculation tests (Fig. 1, Fig. 2) and subsequent experiments.

### 2.2 Reverse transcription–polymerase chain reaction (RT-PCR) and quantitative RT-PCR

Three leaves per plant or condition were ground in liquid nitrogen, and total RNA was extracted with Trizol Reagent (Thermo Fisher Scientific, Inc., Waltham, MA, USA) and treated with RNase-free DNase I (Roche Diagnostics, Basel, Switzerland). cDNA was synthesized from 1 μg of RNA using the modified Moloney murine leukemia virus (MMLV) reverse transcriptase ReverTra Ace® (Toyobo, Osaka, Japan).

Quantitative RT-PCR (qRT-PCR) was performed in an AriaMx real-time PCR system (Agilent Technologies, Santa Clara, CA, USA). Each reaction mixture (25 µl) contained 0.3 mM (each) forward and reverse primers (Supplementary Table S1), 0.2 mM dNTPs, 0.625 U Ex Taq™ DNA polymerase (Takara), SYBR Green (1/800 dilution; Thermo Fisher Scientific), and cDNA corresponding to 50 ng of total RNA. Samples were incubated for 2 min at 95 °C, followed by 40 cycles at 95 °C for 10 s, and at 58 °C and 72 °C for 20 s. The expression of target genes was calculated using the 2^-ΔΔ^*^CT^* method (Livak and Schmittgen, 2001). Tomato *18S* rRNA was used for normalization. The data are presented as means ± SEs from at least three independent experiments.

To construct infectious PVX clones and to clone endogenous genes, PCR was performed as follows. Each reaction mixture (50 µl) contained cDNA corresponding to 100 ng RNA, 0.4 µM of each specific primer pair (Supplementary Table S1), 0.2 mM deoxyribonucleotide triphosphates (dNTPs), 2 mM MgSO_4_, and 0.5 U KOD Plus NEO™ DNA polymerase (Toyobo). PCR mixtures were incubated for 2 min at 94 °C, followed by 30 cycles at 94 °C for 30 s, 60 °C for 30 s, and 68 °C for 180 s. For PR-1a, they were incubated for 2 min at 94 °C, followed by 30 cycles of 94 °C for 30 s, 60 °C for 30 s, and 68 °C for 30 s.

### 2.3 Binary vector constructs

**Fig. 3:**
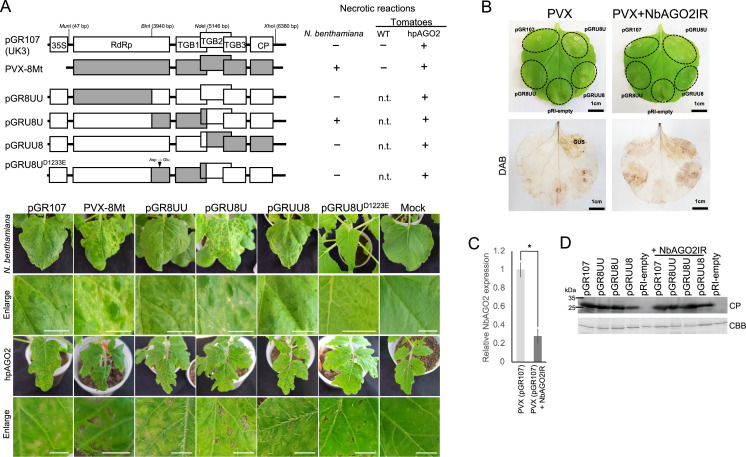
Construction and testing of PVX chimeras*.* (A) Schematic representation of the genomes of PVX-8Mt, pGR107 infectious clone (UK3), and chimeric constructs derived from them. White boxes, ORFs derived from pGR107; gray boxes, ORFs derived from PVX-8Mt. The restriction enzyme sites used are shown above the PVX genome. Symptoms developed in tomato plants (hpAGO2) and *N. benthamiana* inoculated with the indicated viruses or buffer (Mock) were captured at 10 dpi and shown in lower panels. (B) Leaves of *N. benthamiana* agro-infected with pGR107 or PVX chimera viruses in the absence or presence of a construct to silence the *AGO2* gene (NbAGO2IR). At 5 dpi, images were captured (upper panels) and the leaves were stained with DAB (lower panels). pRI-empty was the area of the infiltrated leaf, where an agrobacterium transfomant with a original pRI201 AN binary vector as a control. (C) Relative expression levels of *AGO2* in *N. benthamiana* leaves infiltrated with the indicated viruses. Total RNA was extracted at 6 dpi. The mRNA levels were determined by quantitative RT-PCR and normalized to those of 18S ribosomal RNA. Error bars, standard error of the mean (*n* = 3). *P < 0.05 (Student's *t*-test). (D) Total protein and RNA samples were prepared at 7 dpi from leaf discs infiltrated with the indicated viruses or empty vector (EV). Viral CP was detected with anti-CP antibody to confirm the PVX infection. CBB, Coomassie Brilliant Blue–stained rubisco protein used as a loading control.

The pGR107 expression vector (Jones et al., 1999) and PVX-8Mt were used to construct chimeric viruses. Three chimeric constructs were assembled using restriction enzyme sites present in both PVX cDNA sequences: *Mun*I (bp 47 from the 5′-end of the infectious PVX cDNA; located within the 5′-untranslated region), *Bln*I (bp 3940; located close to the 3′-end of viral RNA-dependent-RNA polymerase (RdRp), *Nde*I (bp 5146; located close to the 3′-end of TGB1, which includes the start codon of TGB2), and *Xho*I (bp 6380; located downstream of the poly(A) sequence in the vector). Each of the three fragments—*Mun*I–*Bln*I (3.9 kb), *Bln*I–*Nde*I (1.2 kb), and *Nde*I–*Xho*I (1.2 kb)—was exchanged between the UK3 and 8Mt strains (Fig. 3A). Site-directed mutagenesis in RdRp at bp 3699, resulting in a substitution from glutamic acid to aspartic acid at amino acid 1233 (PVX-U8U^D1233E^) was carried out as described previouslyas in Yambao et al. (2008). Each chimeric vector was introduced into *Agrobacterium tumefaciens* strain GV3101. To transiently suppress *AGO2* gene expression in *N. benthamiana*, an inverted repeat (IR) was constructed by placing a 520-bp fragment of the *N. benthamiana AGO2* gene (*NbAGO2*: Nbv6.1trP9009 in the *N. benthamiana* database at https://sefapps02.qut.edu.au) in a head-to-head orientation across an intron sequence (Supplementary Fig. S1A) to produce a double-stranded RNA cognate to NbAGO2. The IR sequence was cloned into the NdeI/SalI sites of the binary vector pRI201-AN (Takara, Otsu, Japan) downstream of the CaMV-35S promoter and introduced into A. tumefaciens strain GV3101. To overexpress the S. lycopersicum CCS1 gene (SlCCS1; Solyc08g079830.2 in the tomato genome database at https://solgenomics.net/organism/Solanum_lycopersicum/​genome), the complete coding DNA sequence was cloned into the *Cla*I/*Sal*I sites of the pGR107 and pGRU8U vectors.

### 2.4 Plant growth conditions and agro-infiltration

Tomato and *N. benthamiana* plants were grown in a growth chamber with a 16-h light/8-h dark cycle at 25 °C. For Agroinfiltration assays with *N. benthamiana* plants, *A. tumefaciens* cultures were grown in YEP broth with antibiotics at 28 °C for 48 h. The cells were then pelleted by centrifugation at 3000 rpm for 20 min and re-suspended in MES buffer (10 mM MgCl_2_, 10 mM MES pH 5.7, 100 µM acetosyringone) to a final OD_600_ value of 0.5. Equal volumes of each suspension were then combined.

### 2.5 H_2_O_2_ detection and calculation of disease index

To detect H_2_O_2_, systemic leaves were vacuum infiltrated for 10 min with 3,3′-diaminobenzidine (DAB) solution at 1 mg/mL, and destained by boiling in 100% ethanol for 20 min. H_2_O_2_ was detected from brown coloration owing to the DAB polymerization product formed upon reaction with H_2_O_2_. Disease index was calculated according to Basu et al. (2016). The area of the brown necrotic lesions developed on seedlings and detached leaves were calculated using the ‘ImageJ’ software and represented as the percentage of necrotic area against the whole leaf area.

### 2.6 Northern and western blotting assays

Northern blotting was performed as in Jeon et al. (2017). Upper uninoculated leaves were harvested from each plant at 10, 15, and 20 days post-inoculation (dpi) and total RNA was extracted in Trizol Reagent. The extracts were heated at 65 °C for 15 min in RNA denaturation buffer (0.9 M disodium phosphate, 0.1 M monosodium phosphate, 37% formaldehyde, 0.05% formamide), loaded onto 1.4% agarose gels containing 5% formaldehyde and 1 × 3-(N-morpholino) propanesulfonic acid buffer and run at 100 V for 30 min. Following transfer to a nylon membrane (Hybond-N; GE Healthcare, Chicago, IL, USA), hybridization with digoxigenin (DIG)-labeled probes (Roche Diagnostics) was performed to detect the 3′-terminal regions of PVX genome segments. Chemiluminescence signals were detected and quantified with CDP-star substrate (Millipore Sigma, St. Louis, MO, USA) on a LAS-4000 mini-imaging system (GE Healthcare). sRNA and miRNA were also detected by northern blot assay. Total RNA (∼10 µg) was separated in 12% polyacrylamide gels containing 8 M urea and 0.5 × Tris–boric acid–EDTA (TBE) buffer and run at 200 V for 2 h, transferred to a nylon membrane by a semidry method at 10 V for 1 h, and analyzed by hybridization with a DIG-PVX-cRNA or DIG-miR398a-3p probe (Suzuki et al., 2019).

Western blotting was performed as described in Nakahara et al. (2012) and Jeon et al. (2017). Heat-denatured crude extracts with SDS were separated by electrophoresis in 12% sodium dodecyl sulfate–polyacrylamide gels containing Bis-Tris using Tris-glycine electrode buffer, followed by electro-transfer onto a polyvinylidene difluoride membrane. Antibody raised in rabbit against PVX coat protein (CP) (Japan Plant Protection Association) and anti-Flag-tag monoclonal antibody (Wako, Japan) to detect SlCCS1 were used as primary antibodies at a 1:1000 dilution; alkaline phosphatase–conjugated goat anti-rabbit (Thermo Fisher Scientific) and anti-mouse immunoglobulin G (Bio-Rad, USA) were used as secondary antibodies. Chemiluminescence signals were detected with the CDP-Star substate in the LAS-4000 mini-imaging system.

### 2.7 Deep sequencing of PVX-derived small RNAs

Tomato plants were mock-infected (10 mM phosphate buffer only) or infected with PVX. At 15 dpi, total RNA was isolated from 1 g of leaf tissue in Trizol Reagent. Mixture of total RNAs from three independent tomato plants was used to construct sRNA libraries (1.5 µg of total RNA per library). For small RNA sequencing library preparation, we utilized the NEBNext Multiplex Small RNA Library Prep Set following the provided protocol. After adapter ligation and reverse transcription, a total of 18 PCR cycles were performed. The resulting libraries were subjected to quality control analysis. The pooled libraries were subsequently sequenced using Illumina HiSeq. We obtained around 10 million reads per sample. *S. lycopersicum* ncRNA (rRNA, snRNA, snoRNA, tRNA) sequences were obtained from the RNA sequence collection database, Rfam (Kalvari et al., 2021), provided by the European Bioinformatics Institute. All mature miRNA sequences were obtained from miRBase (Griffiths-Jones et al., 2006) and previous studies (Arazi and Khedia, 2022). The genome data and annotation data SL3.0 for *S. lycopersicum* was downloaded from the Sol Genomics Network: GCA_000188115.3. Low-quality reads and adapter sequences were removed from the four samples of small RNA-seq data using Trim Galore! (Babraham Bioinformatics - Trim Galore! https://www.bioinformatics.babraham.ac.uk/projects/trim_galore/) provided by the Babraham Institute. The 5′ adapter sequence was NNNN, and the 3′ adapter sequence was NNNNTGGAATTCTCGGGTGCCAAGG. The miRNA reference sequences were compiled into a multi-fasta format by merging all miRNA sequences and removing duplicated sequences using CD-HIT. (Fu et al., 2012) During this process, the sequence name from miRBase was given priority for duplicate sequences in the header of the fasta file. The annotation file for SL3.0 was also checked to remove any illegal formats in the strand and gene column. The ncRNA sequences obtained from Rfam were used as reference sequences for mapping small RNA-seq data using Bowtie/1.2.3 (Langmead et al., 2009). The sam format was converted to the bam format, and unmapped reads were extracted using samtools/1.16.1 (Danecek et al., 2021). Finally, the bam format was converted back to the fastq format using the SamToFastq (McKenna et al., 2010) package included in picard-tools/2.25.4 (Picard, http://broadinstitute.github.io/picard/). The converted fastq files were mapped to the reference miRNA sequences using Bowtie/1.2.3 (Langmead et al., 2009). The read count was performed using the samtools-idxstats module of samtools/1.16.1 (Danecek et al., 2021). All read count data was adjusted by adding 0.125, and then converted to RPM (Reads per million) as a relative value per 1 million reads. An MA plot was then generated.

## 3 Results

### 3.1 Systemic necrosis in *AGO2*-silenced tomato and *N. benthamiana* plants infected with PVX

Consistent with our previous observation (Kwon et al., 2020), *AGO2*-silenced transgenic tomato plants (hpAGO2) inoculated with PVX-UK3 developed necrotic symptoms (Fig. 1A). In hpAGO2 plants, necrotic spots began to develop on the second upper leaf from the inoculated leaf at 3 dpi, and necrotic symptoms spread systemically at 15 dpi. When inoculated with PVX-8Mt, which is a necrotic strain to tobacco plants, including *N. benthamiana* (Fig. 1C), hpAGO2 plants showed more prominent necrotic symptoms than those infected with PVX-UK3, whereas wild-type tomato plants infected with either strain showed no necrotic symptoms. DAB staining detected the accumulation of H_2_O_2_ (Fig. 1B) along with necrotic symptoms.

To investigate whether the difference in necrotic reactions was associated with the extent of PVX multiplication, we analyzed genomic RNA accumulation by northern blotting. After infection with PVX-UK3, viral RNA accumulation was higher in hpAGO2 than in wild-type tomato plants at all tested time points (Fig. 2A). Similarly, viral RNA accumulation was higher in PVX-8Mt-infected hpAGO2 than in wild-type plants (Fig. 2B). These results show that the necrosis symptoms induced in hpAGO2 by PVX infection were related to increased viral accumulation. However, more severe necrosis caused by PVX-8Mt could not be ascribed to increased viral accumulation since viral RNA accumulation in PVX-8Mt-infected hpAGO2 plants was lower than in PVX-UK3-infected plants (Fig. 2B). Because necrotic symptoms generally involve induction of salicylic acid signaling (Nie 2006; Jovel et al., 2011; Murphy et al., 2020), we investigated the expression of the gene encoding PR-1a, which is a typical index gene for salicylic acid signaling. As expected, strong induction of the PR-1a gene matched the development of necrotic symptoms whereas PR-1a induction was much lower in infected wild-type tomato plants (Fig. 2B). Virus-derived small interfering RNAs also accumulated to a greater extent in hpAGO2 than in wild-type plants (Fig. 2C).

### 3.2 PVX RdRp is a viral determinant of necrotic symptoms in *N. benthamiana*

To identify the region of the viral genome responsible for the higher virulence of PVX-8Mt than that of PVX-UK3, we constructed cDNA chimeras between the two strains (Fig. 3A). Unfortunately, the hpAGO2 tomato plants exhibited comparable necrotic symptoms when inoculated with any of the chimeric PVX variants (Fig. 3A lower panels). We were unable to map the viral genomic region responsible for the more prominent necrotic symptoms observed in hpAGO2 tomato plants infected with PVX-8Mt. Notably, only the PVX-U8U chimera induced necrosis in systemic leaves of agroinfected *N. benthamiana* plants (Fig. 3A) and agroinfected area of a *N. benthamiana* leaf (Fig. 3B, left); this chimera carried the N terminal part of the viral replicase and the full TGB1 ORF derived from PVX-8Mt (Fig. 3A). Consistently, the agroinfection area of PVX-U8U was stained with DAB, indicating H_2_O_2_ accumulation. These results indicate that the region of PVX-U8U originated from PVX-8Mt was responsible for the necrotic reaction in *N. benthamiana*. As a positive control, we investigated the reactions of *N. benthamiana* to these chimeric PVXs under conditions of AGO2 gene silencing by co-infiltrating an *Agrobacterium* transformant carrying the NbAGO2 IR, which effectively suppressed *AGO2* expression (Fig. 3C). All PVX chimeras caused necrosis with accumulations of H_2_O_2_ (Fig. 3B) and viral CP (Fig. 3D), similar to the necrotic symptoms observed in hpAGO2 tomato plants infected with these chimeric PVXs.

By comparing the nucleotide sequence of the 3940–5146 bp region of PVX-8Mt with that of PVX-UK3, we found two non-synonymous substitutions in the C-terminal part of RdRp. Among them, valine into isoleusine at aa position 1169 and glutamic acid into aspartic acid at aa position 1233 (E1233D), E1233D in RdRp has been reported to be critical for PVX necrotic symptom in tobacco (Kagiwada et al., 2005). To examine whether the necrotic symptoms by PVX-8Mt was attributable to E1233D, we created a point mutant of the chimera (PVX-U8U^D1233E^). When inoculated into *N. benthamiana*, PVX-U8U^D1233E^ did not cause necrosis but PVX-U8U did (Fig. 3A), suggesting that viral RdRp is responsible for the necrotic symptoms by PVX-8Mt in *N. benthamiana* plants.

### 3.3 Down regulation of the copper chaperone CCS1 was associated with systemic necrosis in AGO2-silenced tomato plants

**Fig. 4:**
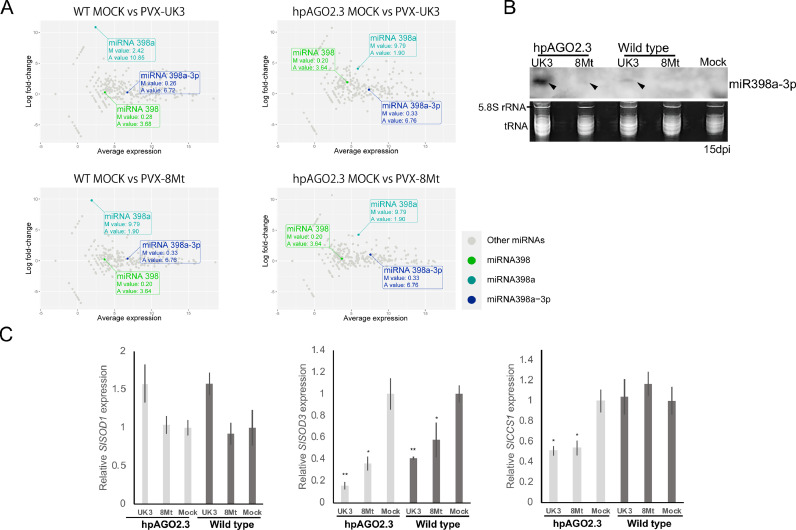
Expression levels of *miR398*s and SODs in healthy and PVX-infected tomato leaves with or without showing necrosis. (A) Small RNA sequencing of wild-type and hpAGO2 leaves infected with PVX-UK3 or PVX-8Mt or infiltrated with buffer (Mock). M, log fold change; A, average abundance. (B) Northern-blot hybridization analysis of *miR398*a-3p performed as in Fig. 3E. tRNA and 5S RNA, loading controls stained with EtBr. (C) Relative levels of the *SlSOD1, SlSOD3*, and *SlCCS1* mRNAs in hpAGO2 and wild-type tomato plants under infection with PVXs. The mRNA levels were determined by quantitative RT-PCR. *P < 0.05, **P < 0.01 vs. mock-infected plants (Student's *t*-test).

Next, we examined the possible host factors involved in the induction of systemic necrosis in AGO2-silenced tomato plant. Recently, systemic necrosis was observed in DCL2,4-silenced tomato plants (cv. moneymaker) infected with PSTVd (Suzuki et al., 2019). The study showed that induction of *miR398*s and downregulation of their target gene encoding copper chaperone for SOD (CCS) 1 are associated with systemic necrosis. In *A. thaliana, miR398, miR398*a, and *miR398*a-3p negatively regulate SODs and CCS1, which normally scavenge harmful O_2_^−^ (Sunkar et al., 2006; Feng et al., 2016; Arazi and Khedia 2022). To examine the possible involvement of host *miR398*s and their targets, SODs and CCS1, in systemic necrosis induced by PVX infection, we investigated their expression levels in PVX-infected hpAGO2 and wild-type tomato plants. Small RNA sequencing showed higher abundance of *miR398*s reads in hpAGO2 infected with either PVX-UK3 or −8Mt than in mock-infected hpAGO2 plants (Fig. 4A, Supplementary Table S2). We here note that only the abundance of *miR398*a reads drastically increased in wild-type tomatoes infected with either PVX strain, although its basal level was lower in wild-type than in hpAGO2 tomatoes. We also showed that PVX infection altered the expression levels of many miRNAs (Fig. 4A). The number of microRNAs with increased accumulation levels was greater than the number with decreased accumulation levels, and this trend was more pronounced in hpAGO2 plants compared to the wild-type. In PVX-UK3 infected wild-type plants, 211 miRNAs were upregulated and 129 miRNAs were downregulated (Supplementary Table S2). In PVX-UK3 infected hpAGO2 plants, 246 miRNAs were upregulated and 97 miRNAs were downregulated (Supplementary Table S2). Similar results were obtained with PVX-8Mt. Since the basal level of *miR398a*-3p was highest in both wild-type and hpAGO2 plants and it would be easier to detect than other *miR398*s, we performed northern blotting with an oligonucleotide probe for *miR398*a-3p. At 15 dpi, we detected higher accumulation of *miR398*a-3p in hpAGO2 tomato plants infected with both PVXs than in wild-type tomato plants infected with both PVXs, confirming the induction of miR398a-3p in hpAGO2 plants infected with PVXs (Fig. 4B).

We confirmed the presence of the target sequences of *miR398, miR398*a, and *miR398*a-3p in the *SlSOD1, SlSOD3*, and *SlCCS1* genes (Supplementary Fig. S2) but not confirmed in *SlSOD2* and thus examine the expression levels of *SlSOD1, SlSOD3* and *SlCCS1* by real-time PCR (Fig. 4C). Among the *SlSOD1, SlSOD3*, and *SlCCS1* transcripts, only the reduced expression level of *SlCCS1* was consistent with the induction of systemic necrosis symptoms in the upper leaves (Fig. 4C): at 15 dpi, *SlCCS1* was downregulated by almost half in hpAGO2 plants inoculated with either PVX strain, which showed necrosis, but not in infected wild-type plants, which showed no necrotic symptoms. *SlSOD3* was downregulated both in hpAGO2 and in wild-type plants infected with either PVX strain (Fig. 4C). *SlSOD1* expression was not significantly different among the samples (Fig. 4C). Downregulation of *SlCCS1* and *SlSOD3* was consistent with increased accumulation of *miR398s* in PVX-infected WT and hpAGO2 plants (Fig. 4A and B), implying *miR398s* regulate their expression. However, the expression patterns of *SlCCS1* and *SlSODs,* particularly *SlSOD1*, could not be fully explained by *miR398* regulation, indicating the involvement of additional mechanisms. Since AGO1 is involved in miRNA biogenesis (Jung et al., 2009; Xie et al., 2015; Zhang et al., 2020; Giudicatti et al., 2021; Liang et al., 2022), we examined the levels of the *SlAGO1* transcripts. *SlAGO1* transcript accumulation was not significantly different between hpAGO2 and wild-type plants regardless of PVX infection and, as expected (Kwon et al., 2020), the *AGO2* gene was silenced in hpAGO2 plants, regardless of PVX infection (Supplementary Fig. S3).

### 3.4 CCS1 expression through viral vectors alleviates systemic necrosis induced by infection of AGO2-silenced tomato plants with PVX

**Fig. 5:**
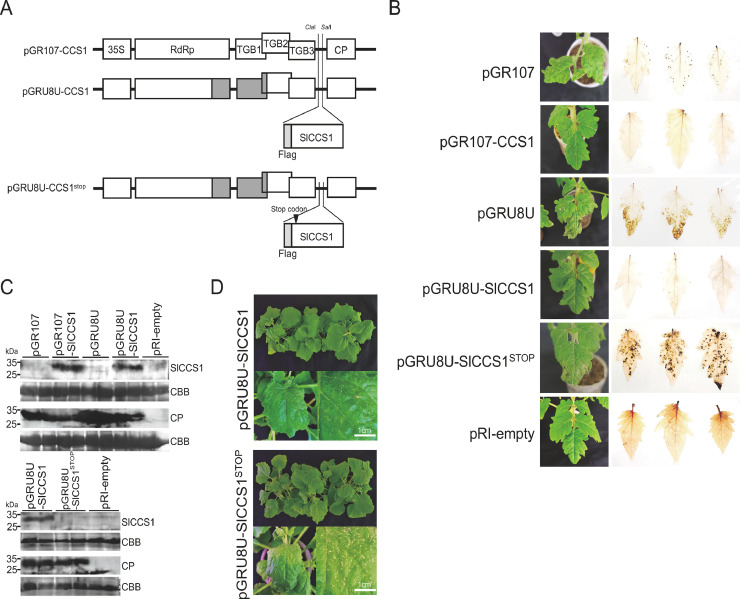
Expression of SlCCS1 together with PVX suppresses necrosis in hpAGO2 tomato and *N. benthamiana* plants. (A) Complete *SlCCS1* coding sequence fused to a FLAG tag–encoding sequence or the same sequence with an introduced stop codon was cloned into the *Cla*I and *Sal*I sites of the PVX infectious clones. (B) Symptoms developed in hpAGO2 plants inoculated with the indicated PVXs. Wild-type Moneymaker plants were also infiltrated with the pRI201 AN empty vector (pRI-empty) as a control. Images were captured and the upper leaves were stained with DAB at 10 dpi. (C) Detection of SlCCS1 and viral CP by western blotting. Total protein was extracted from leaves of plants shown in (B) at 10 dpi. The CBB-stained gel was used as a loading control. (D) Symptoms developed in *N. benthamiana* plants inoculated with the indicated PVXs at 15 dpi.

To further investigate whether the downregulation of *SlCCS1* by *miR398*s is responsible for induction of systemic necrosis. In AGO2-silenced plants, we inserted the cDNA of the *SlCCS1* ORF into pGR107 and pGRU8U (necrotic pathotype) and inoculated them into hpAGO2 plants (Fig. 5A)*.* As expected, necrotic symptoms and spots were observed in hpAGO2 plants infected with both PVX-UK3 and PVX-U8U. However, the necrotic symptoms were attenuated in hpAGO2 plants infected with either PVX expressing *SlCCS1* (Fig. 5B). Western blotting confirmed the expression of Flag-tagged *SlCCS1* from the recombinant PVX at 15 dpi (Fig. 5C, upper panels). The hpAGO2 plants inoculated with the U8U chimera with a stop codon in the *SlCCS1* ORF (Fig. 5A) showed severe necrotic symptoms (Fig. 5B), indicating that symptom attenuation was caused by the ectopically expressed *SlCCS1* protein; no *SlCCS1* protein was produced from this chimera (Fig. 5C, lower pannels). These results indicate that downregulation of *SlCCS1* is responsible for systemic necrosis in hpAGO2 plants infected with PVX. We then examined whether *SlCCS1* was also involved in necrotic symptoms in *N. benthamiana* infected with PVX-8Mt and PVX-U8U (Fig. 3). As a result of inoculation test, PVX-U8U expressing SlCCS1 induced less severe necrotic symptoms than PVX-U8U with *SlCCS1* stop (Fig. 5D), suggesting that SlCCS1 is also involved in induction of necrotic symptoms in *N. benthamiana*.

## 4 Discussion

This study examined the mechanisms underlying exacerbated symptoms (systemic necrosis) in AGO2-silenced plants infected with PVX. These symptoms were associated with increased accumulation of viral genomic RNA and ROS (Fig. 1). We also showed that the expression of *miR398*s and one of their targets, *CCS1*, was associated with these symptoms. Gene expression using PVX infectious vectors proved that *CCS1*, chaperone for SODs, was involved in the necrotic symptoms.

In plants, defense reactions (such as HR) to pathogen infection often lead to elevated levels of ROS, such as superoxide and H_2_O_2_ (Lamb and Dixon, 1997). Comprehensive global transcriptome and metabolome analyses suggest that viroid infections trigger ROS biogenesis, plant immune responses, and hormonal signaling pathways and associated genes, such as those for the PR proteins (Xia et al., 2017; Zheng et al., 2017). Infection with various viruses is associated with ROS production in plants; for example, members of the genus *Potyvirus* cause systemic necrosis with accumulation of ROS (Nie 2006; Kim et al., 2008; Yambao et al., 2008; Atsumi et al., 2009; Haikonen et al., 2013; Peng et al., 2013; Hisa et al., 2014; Gaguancela et al., 2016; Otulak-Kozieł et al., 2019; Yang et al., 2020; Al-Mokadem et al., 2022; Otulak-Kozieł et al., 2023) and, regardless of necrosis, infection with plum pox virus increases the level of ROS in pea (Desikan et al., 1996). The levels of H_2_O_2_, superoxide, and cucumber mosaic virus (CMV)-CP are intercorrelated with symptom development in CMV-infected *Nicotiana glutinosa* (Lei et al., 2016; Shang et al., 2018). Similarly, H_2_O_2_ is produced in *N. glutinosa* and *N. tabacum* cv. Xanthi-nc infected with the incompatible tobacco mosaic virus (TMV) or tomato mosaic virus (ToMV) (Madhusudhan et al., 2009). Similarly, we found that ROS accumulated in necrotic tissues of hpAGO2 tomato plants and *N. benthamiana* infected with PVX (Fig. 1B, C).

ROS are toxic and would exacerbate symptoms to some extent, but they are also important signaling molecules, together with Ca^2+^, for immune responses (Qi et al., 2017; Marcec et al., 2019; Khan et al., 2023), and we considered the possibility of symptom exacerbation by ROS-mediated induction of defense reactions. We previously showed in the pathosystem of clover yellow vein virus (ClYVV) and susceptible pea plants that activation of salicylic acid signaling by an exogenous salicylic acid analogue, BTH, enhanced ClYVV virulence (Atsumi et al., 2009). The mRNA level of *PR-1a* increased in *AGO2*-knockdown plants but not wild-type tomato plants infected with PVX (Fig. 2B). As elevated ROS levels enhance salicylic acid signaling and related defense genes (Uknes et al., 1992; Maleck and Dietrich 1999), elevated ROS in *AGO2*-knockdown plants infected with PVX may have contributed to systemic necrosis by enhancing salicylic acid signaling. In *N. benthamiana*, HR and host factors involved in HR mediate systemic necrosis in plants infected with *Plantago asiatica* mosaic virus (PlAMV) (Komatsu et al., 2011; Komatsu 2013).

AGO2 is critical for RNA silencing-mediated defense against PVX in Arabidopsis; an AGO2 mutant but not Col-0 can be infected systemically by PVX (Jaubert et al., 2011). Therefore, *AGO2* knockdown would weaken RNA silencing defense against PVX, resulting in increased PVX multiplication. Elevated ROS might be another reason for the increased multiplication of PVX in *AGO2*-knockdown tomato plants, as ROS reportedly enhance multiplication of viruses, including red clover necrotic mosaic virus and CMV (Hyodo et al., 2017; Jeon et al., 2017; Murota et al., 2017; Kanodia and Miller, 2022).

Suzuki et al. (2019) found that infection of DCL2- and -4-silenced tomato plants with PSTVd leads to systemic necrosis associated with induction of *miR398*s and a decrease in the expression of their target gene *CCS1*. Consistently, in this study, systemic necrosis in *AGO2*-knockdown tomato plants infected with PVX was also associated with the induction of *miR398*s and a decrease in *CCS1* transcript level. However, the mechanism by which *miR398*s are upregulated in PVX-infected plants are not fully understood. Although miRNAs are processed by AGO1 (Jung et al., 2009; Xie et al., 2015; Zhang et al., 2020; Giudicatti et al., 2021; Liang et al., 2022), *SlAGO1a* and *b* transcript levels were not significantly affected by PVX infection (Supplementary Fig. S3). Small RNA-seq data showed that there were more miRNAs, including *miR398*s, whose levels were increased by PVX infection than those whose levels were decreased as described the result section with Fig. 4A. This non-specific induction of miRNAs may at least in part contribute to the elevated levels of *miR398*s; they are expressed in response to various stresses and inhibit the expression of cytosolic and chloroplastic SODs (Juszczak and Baier, 2012) and CCSs for SODs (i.e., CCS1) (Beauclair et al., 2010).

SODs play a vital role in converting superoxide to H_2_O_2_ and molecular oxygen and thus reduce oxidative stress in plant cells. As two members of SODs, SOD1 and SOD2 function in the ROS scavenging system and protect plants from oxidative stress. The expression of both SOD1 and SOD2 is induced under oxidative stress conditions due to the down-regulation of miR398 (Sunker et al., 2006). Also, the other study reported that miR398 directs degradation of the CCS1 transcript and is therefore a target for miR398 (Li et al., 2010). This report support to correlation between the miR398 and SODs.

Using the PVX vector expressing *CCS1*, we proved that reduction of *CCS1* expression is responsible for systemic necrosis (Fig. 5). In silico analysis with psRNATarget suggested that *miR398*s can bind to and target *SlSOD1, SlSOD3*, and *SlCCS1* transcripts (Supplementary Fig. S2). The *SlSOD3* mRNA levels but not *SlSOD1* decreased significantly when plants were infected with PVX (Fig. 4C), perhaps owing to elevated *miR398*s. A decrease in O_2_^−^ scavenging by SODs would increase toxicity and elicitation of defense signaling, resulting in systemic necrosis. The production of ROS is an important defense reaction against pathogens that is rapidly induced upon recognition of a pathogen attack. Recent data from comprehensive and global transcriptome and metabolome analyses suggested that viral infections trigger plant immune responses and result in the activation of various signaling pathways and associated activities, such as MAPK3, PR1, 1,3-beta-glucanase, and ROS biogenesis (Chávez-Calvillo et al., 2016; Lee et al., 2018; Frąckowiak et al., 2019). On the other hand, ROS are harmful substances that can cause significant damage to cell structures. Therefore, the proper management of ROS production and scavenging is very important when plants are protecting themselves against pathogen attacks. The results presented in this study identified several key factors (i.e., *miR398s, CCS1*, and ROS) in tomato plants which are responsible for causing necrosis, one of the most typical and serious disease symptoms induced by viral infections.

Several plant species accumulate *miR398* upon viral infection, such as *A. thaliana* infected with TMV or oilseed rape mosaic *Tobamovirus* (Bazzini et al., 2011; Hu et al., 2011) and *N. benthamiana* infected with bamboo mosaic virus (BaMV), PVX, potato virus Y (PVY), or tomato yellow leaf curl China virus (TolCNV) (Naqvi et al., 2010; Pacheco et al., 2012; Lin et al., 2022). Although the levels of their targets, SODs, are variable (Naqvi et al., 2010; Pacheco et al., 2012), the control of SODs by elevated *miR398*s appears to occur widely in various virus–plant combinations, resulting in enhanced virus virulence and multiplication.

**Fig. 6:**
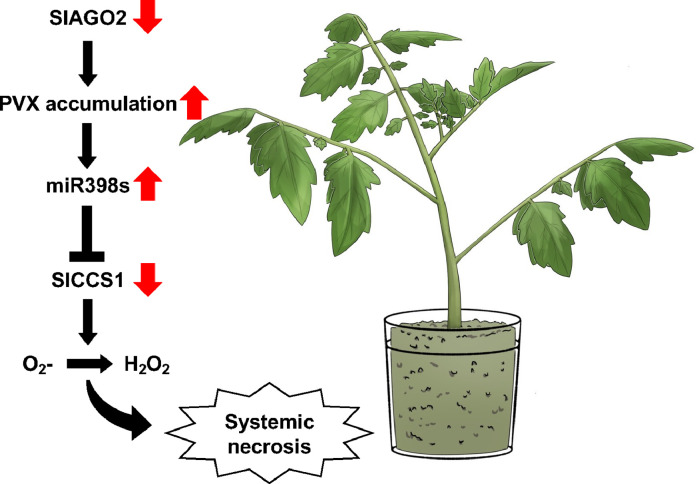
The schematic illustrates our findings regarding the involvement of SlCCS1 in development of necrotic symtoms by PVX infection in AGO2-silenced tomato plants. *miR398*s are induced by PVX accumulation and its target *SlCCS1* transcript is downregulated, affecting ROS scavenging and leading to systemic necrosis.

Overall, this study proved that downregulation of *SlCCS1* is involved in systemic necrosis in *AGO2*-silenced plants. On the basis of our findings, we propose a model of PVX-induced systemic necrosis in AGO2-silenced plants (Fig. 6). Since AGO2 is critical for RNA silencing-mediated defense to PVX, *AGO2* silencing increased PVX multiplication, and higher PVX accumulation may have elicited stronger host reactions, resulting in higher levels of *miR398*s and ROS. Downregulation of SlCCS1 reduced ROS scavenging, leading to systemic necrosis.

## Author statement

No AI technologies (large language models or otherwise) were used in the analysis or preparation of this manuscript.

## Funding acknowledgments

This research was supported by JSPS KAKENHI Grant Numbers JP22H02343 and JP18H02201.

## CRediT authorship contribution statement

**Joon Kwon:** Writing – review & editing, Writing – original draft, Investigation. **Kento Mori:** Investigation, Formal analysis. **Tetsuo Maoka:** Writing – review & editing, Resources. **Teruo Sano:** Writing – review & editing, Resources. **Kenji S. Nakahara:** Writing – review & editing, Writing – original draft, Validation, Supervision.

## Declaration of competing interest

The authors declare that they have no known competing financial interests or personal relationships that could have appeared to influence the work reported in this paper.

## Data Availability

Data will be made available on request.

## Appendix Supplementary materials

Image, application 1

Image, application 2
