# Supplementary material for: Induction of necrosis symptoms by potato virus X in AGO2-silenced tomato plants associates with reduced transcript accumulation of copper chaperon for superoxide dismutase gene
Source: Virus Res. 2024 Jul 18;348:199436. doi: 10.1016/j.virusres.2024.199436 (PMC11315226; doi:10.1016/j.virusres.2024.199436)
Supplement: Supplementary file 2 [file mmc2.pptx]

## Slide 1
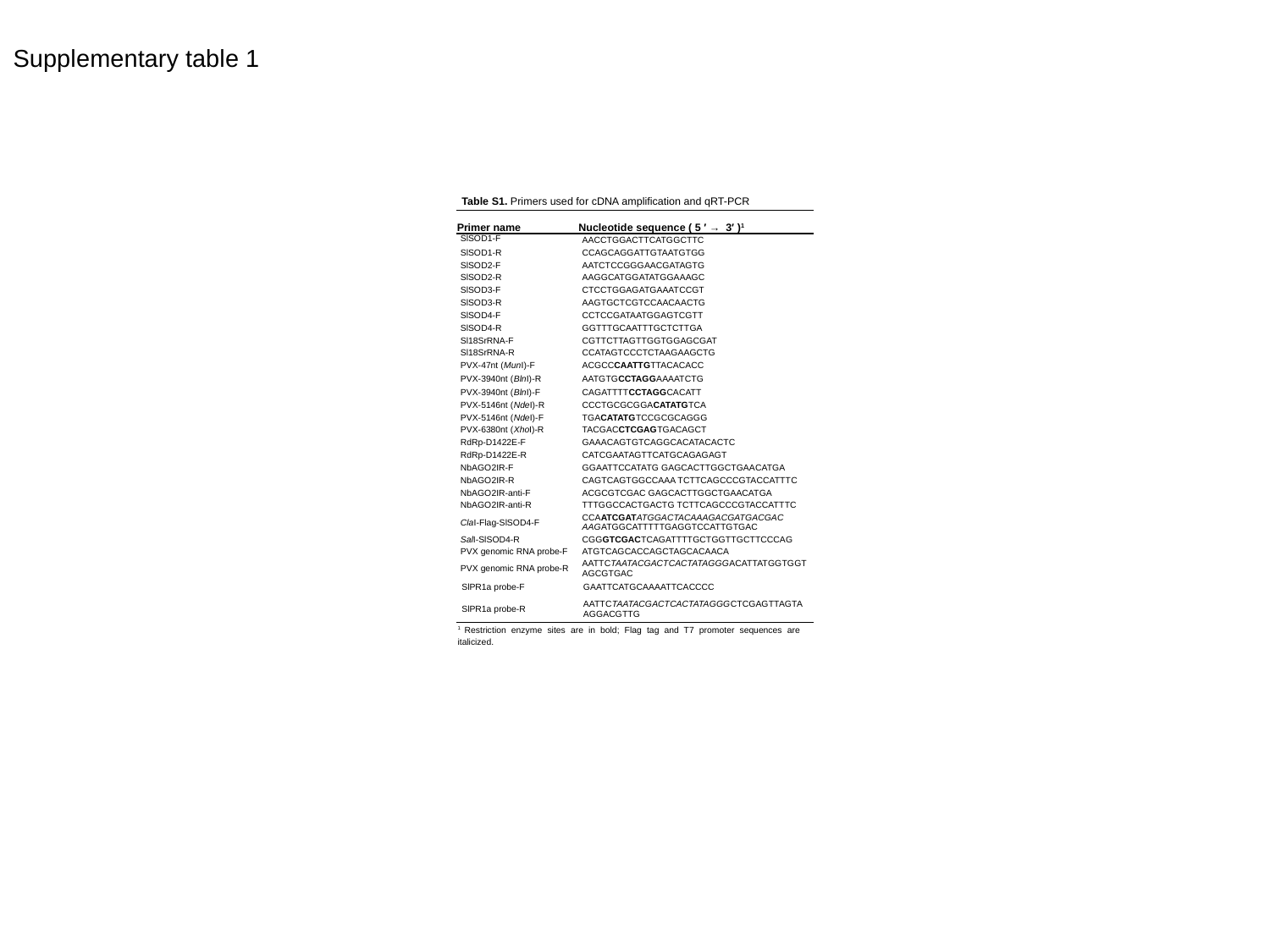

Supplementary table 1
| Table S1. Primers used for cDNA amplification and qRT-PCR | |
| --- | --- |
| Primer name | Nucleotide sequence ( 5 ′ → 3′ )1 |
| SlSOD1-F | AACCTGGACTTCATGGCTTC |
| SlSOD1-R | CCAGCAGGATTGTAATGTGG |
| SlSOD2-F | AATCTCCGGGAACGATAGTG |
| SlSOD2-R | AAGGCATGGATATGGAAAGC |
| SlSOD3-F | CTCCTGGAGATGAAATCCGT |
| SlSOD3-R | AAGTGCTCGTCCAACAACTG |
| SlSOD4-F | CCTCCGATAATGGAGTCGTT |
| SlSOD4-R | GGTTTGCAATTTGCTCTTGA |
| Sl18SrRNA-F | CGTTCTTAGTTGGTGGAGCGAT |
| Sl18SrRNA-R | CCATAGTCCCTCTAAGAAGCTG |
| PVX-47nt (MunI)-F | ACGCCCAATTGTTACACACC |
| PVX-3940nt (BlnI)-R | AATGTGCCTAGGAAAATCTG |
| PVX-3940nt (BlnI)-F | CAGATTTTCCTAGGCACATT |
| PVX-5146nt (NdeI)-R | CCCTGCGCGGACATATGTCA |
| PVX-5146nt (NdeI)-F | TGACATATGTCCGCGCAGGG |
| PVX-6380nt (XhoI)-R | TACGACCTCGAGTGACAGCT |
| RdRp-D1422E-F | GAAACAGTGTCAGGCACATACACTC |
| RdRp-D1422E-R | CATCGAATAGTTCATGCAGAGAGT |
| NbAGO2IR-F | GGAATTCCATATG GAGCACTTGGCTGAACATGA |
| NbAGO2IR-R | CAGTCAGTGGCCAAA TCTTCAGCCCGTACCATTTC |
| NbAGO2IR-anti-F | ACGCGTCGAC GAGCACTTGGCTGAACATGA |
| NbAGO2IR-anti-R | TTTGGCCACTGACTG TCTTCAGCCCGTACCATTTC |
| ClaI-Flag-SlSOD4-F | CCAATCGATATGGACTACAAAGACGATGACGAC AAGATGGCATTTTTGAGGTCCATTGTGAC |
| SalI-SlSOD4-R | CGGGTCGACTCAGATTTTGCTGGTTGCTTCCCAG |
| PVX genomic RNA probe-F | ATGTCAGCACCAGCTAGCACAACA |
| PVX genomic RNA probe-R | AATTCTAATACGACTCACTATAGGGACATTATGGTGGTAGCGTGAC |
| SlPR1a probe-F | GAATTCATGCAAAATTCACCCC |
| SlPR1a probe-R | AATTCTAATACGACTCACTATAGGGCTCGAGTTAGTAAGGACGTTG |
1 Restriction enzyme sites are in bold; Flag tag and T7 promoter sequences are italicized.

## Slide 2
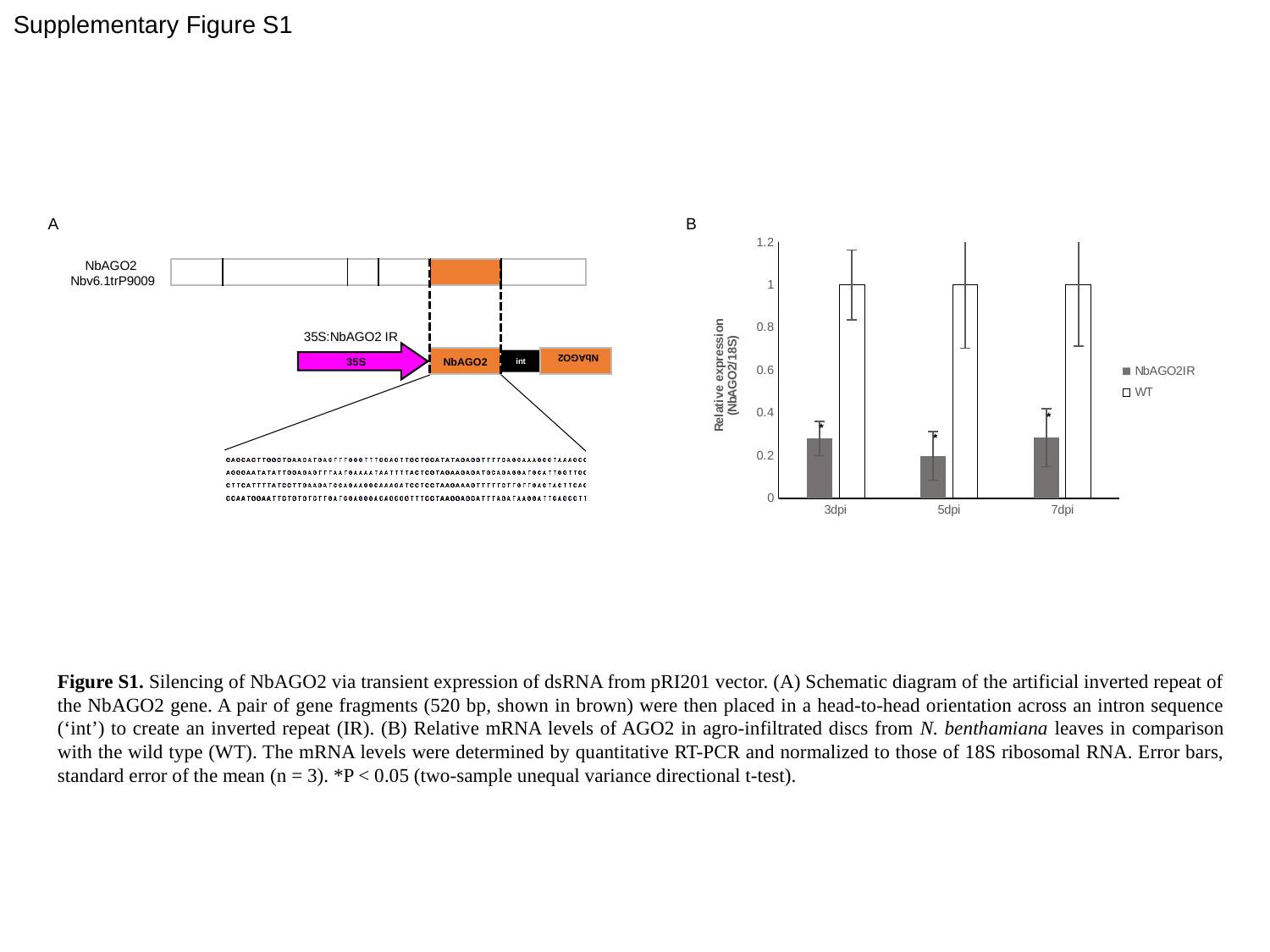

Supplementary Figure S1
A
B
### Chart
| Category | NbAGO2IR | WT |
|---|---|---|
| 3dpi | 0.27972901325554206 | 1.0 |
| 5dpi | 0.19844863323692427 | 1.0 |
| 7dpi | 0.2845505060161001 | 1.0 |*
*
*
NbAGO2
Nbv6.1trP9009
| | | | | | |
| --- | --- | --- | --- | --- | --- |
35S:NbAGO2 IR
35S
| |
| --- |
| NbAGO2 |
| --- |
NbAGO2
int
Figure S1. Silencing of NbAGO2 via transient expression of dsRNA from pRI201 vector. (A) Schematic diagram of the artificial inverted repeat of the NbAGO2 gene. A pair of gene fragments (520 bp, shown in brown) were then placed in a head-to-head orientation across an intron sequence (‘int’) to create an inverted repeat (IR). (B) Relative mRNA levels of AGO2 in agro-infiltrated discs from N. benthamiana leaves in comparison with the wild type (WT). The mRNA levels were determined by quantitative RT-PCR and normalized to those of 18S ribosomal RNA. Error bars, standard error of the mean (n = 3). *P < 0.05 (two-sample unequal variance directional t-test).

## Slide 3
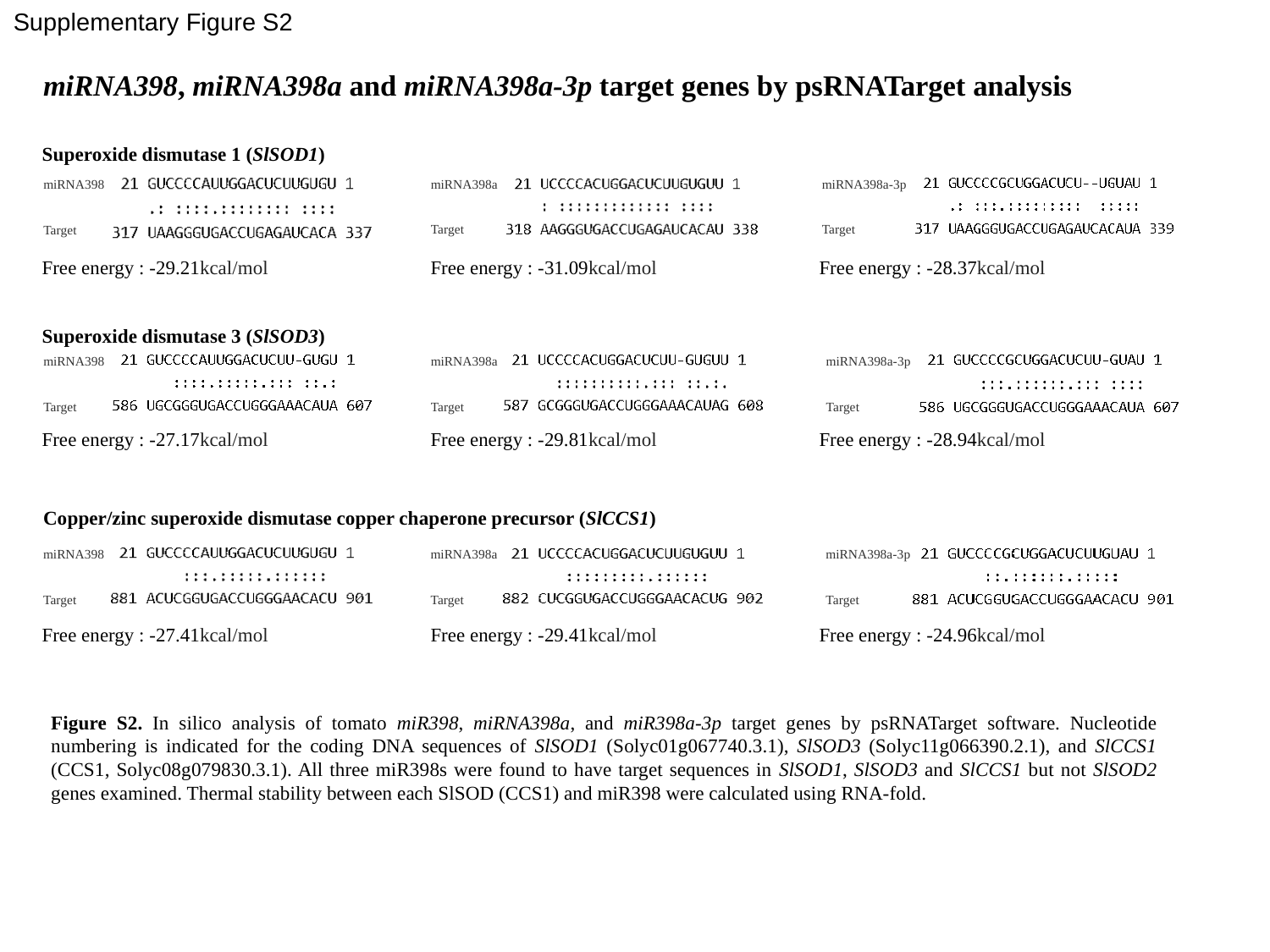

Supplementary Figure S2
miRNA398, miRNA398a and miRNA398a-3p target genes by psRNATarget analysis
Superoxide dismutase 1 (SlSOD1)
miRNA398a
miRNA398a-3p
miRNA398
Target
Target
Target
Free energy : -29.21kcal/mol
Free energy : -31.09kcal/mol
Free energy : -28.37kcal/mol
Superoxide dismutase 3 (SlSOD3)
miRNA398
miRNA398a
miRNA398a-3p
Target
Target
Target
Free energy : -27.17kcal/mol
Free energy : -29.81kcal/mol
Free energy : -28.94kcal/mol
Copper/zinc superoxide dismutase copper chaperone precursor (SlCCS1)
miRNA398
miRNA398a
miRNA398a-3p
Target
Target
Target
Free energy : -27.41kcal/mol
Free energy : -29.41kcal/mol
Free energy : -24.96kcal/mol
Figure S2. In silico analysis of tomato miR398, miRNA398a, and miR398a-3p target genes by psRNATarget software. Nucleotide numbering is indicated for the coding DNA sequences of SlSOD1 (Solyc01g067740.3.1), SlSOD3 (Solyc11g066390.2.1), and SlCCS1 (CCS1, Solyc08g079830.3.1). All three miR398s were found to have target sequences in SlSOD1, SlSOD3 and SlCCS1 but not SlSOD2 genes examined. Thermal stability between each SlSOD (CCS1) and miR398 were calculated using RNA-fold.

## Slide 4
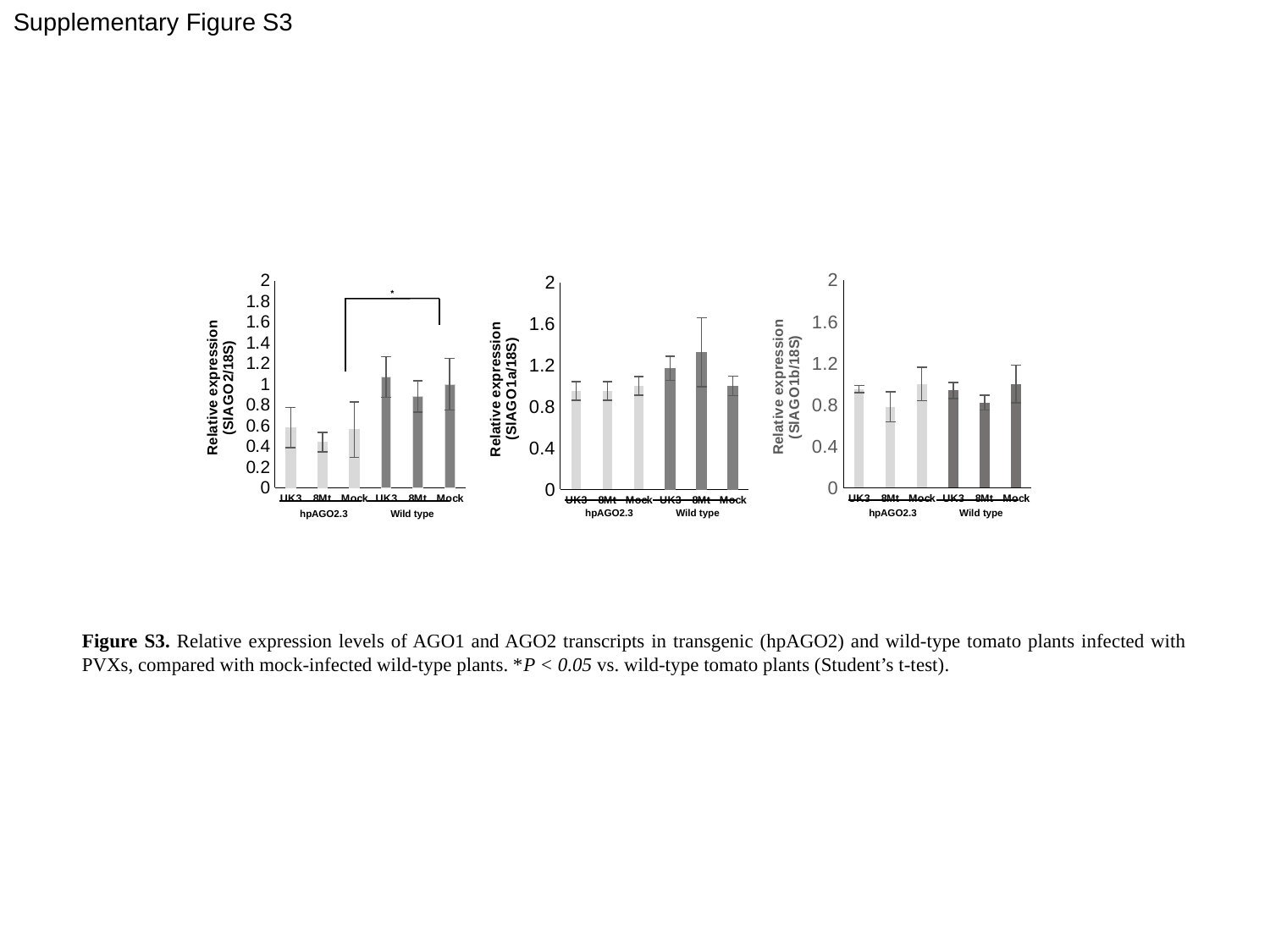

Supplementary Figure S3
### Chart
| Category | AGO1b |
|---|---|
| UK3 | 0.9506342780517737 |
| 8Mt | 0.7801175824656307 |
| Mock | 1.0 |
| UK3 | 0.9374436525458764 |
| 8Mt | 0.8190740011869108 |
| Mock | 1.0 |
### Chart
| Category | AGO2 |
|---|---|
| UK3 | 0.5810906241644096 |
| 8Mt | 0.44126098391516216 |
| Mock | 0.5612479369159057 |
| UK3 | 1.0693600194958535 |
| 8Mt | 0.8817654473961006 |
| Mock | 1.0 |
### Chart
| Category | AGO1a |
|---|---|
| UK3 | 0.95057046509242 |
| 8Mt | 0.95057046509242 |
| Mock | 1.0 |
| UK3 | 1.1681823014496049 |
| 8Mt | 1.3243127023643029 |
| Mock | 1.0 |*
 hpAGO2.3 Wild type
 hpAGO2.3 Wild type
 hpAGO2.3 Wild type
Figure S3. Relative expression levels of AGO1 and AGO2 transcripts in transgenic (hpAGO2) and wild-type tomato plants infected with PVXs, compared with mock-infected wild-type plants. *P < 0.05 vs. wild-type tomato plants (Student’s t-test).
